# Multimodality cardiac imaging of a left ventricular papillary fibroelastoma: a case report

**DOI:** 10.1186/s13104-016-2323-9

**Published:** 2017-01-07

**Authors:** Rajat Sharma, Mehrdad Golian, Pallav Shah, Davinder S. Jassal, Nasir Shaikh

**Affiliations:** 1 Section of Cardiology, Department of Internal Medicine, Max Rady College of Medicine, Rady Faculty of Health Sciences, Rm Y3531, Bergen Cardiac Care Centre, St. Boniface Hospital, University of Manitoba, 409 Tache Avenue, Winnipeg, Manitoba Canada R2H 2A6; 2 Section of Cardiac Surgery, Department of Surgery, Max Rady College of Medicine, Rady Faculty of Health Sciences, Rm Y3531, Bergen Cardiac Care Centre, St. Boniface Hospital, University of Manitoba, 409 Tache Avenue, Winnipeg, Manitoba Canada R2H 2A6; 3 Department of Radiology, Max Rady College of Medicine, Rady Faculty of Health Sciences, Rm Y3531, Bergen Cardiac Care Centre, St. Boniface Hospital, University of Manitoba, 409 Tache Avenue, Winnipeg, Manitoba Canada R2H 2A6; 4 Institute of Cardiovascular Sciences, St. Boniface Albrechtsen Research Centre, University of Manitoba, 351 Tache Avenue, Winnipeg, Manitoba Canada R2H 2A6

**Keywords:** Papillary fibroelastoma, Echocardiography, Cardiac MRI

## Abstract

**Background:**

In the setting of an acute myocardial infarction (AMI), although the most common etiology of a left ventricular (LV) mass identified on multimodality cardiovascular imaging is a thrombus, other possibilities including a vegetation or tumor should be entertained within the differential diagnosis.

**Case presentation:**

We describe a case of a 43-year-old Caucasian female post AMI diagnosed with a mid-cavitary mass within the LV. Although echocardiography and cardiovascular MRI (CMR) suggested that the mass was a thrombus, given the context of the recent AMI, exploration and surgical excision was completed by the surgeon due to the potential for the mass to embolize.

**Conclusion:**

The final diagnosis of a papillary fibroelastoma was unique due to its unusual location and large size within the LV cavity. This unique case demonstrates shortcomings of multimodality cardiac imaging in the diagnosis of an atypical mass and the importance of obtaining tissue when clinically safe and feasible.

## Background

Primary tumors of the heart are infrequent with an overall incidence of 0.02% based on autopsy series [1]. With the routine use of noninvasive imaging, cardiac tumors are often found incidentally. However, a left ventricular (LV) mid-cavitary mass is rarely seen and can challenge a clinicians’ diagnostic ability. The diagnosis relies on multimodality cardiovascular imaging, including echocardiography, cardiac computed tomography (CCT), and cardiovascular magnetic resonance (CMR). The etiology of a LV mass can be determined by its location, attachment, mobility, enhancement, and vascularity.

In the setting of an acute myocardial infarction (AMI), a LV mass is likely to represent a thrombus. Approximately 7% of patients with an anterior AMI develop a LV thrombus [2, 3], which is considerably more prevalent than primary cardiac tumors. Nonetheless, it is important to have a broad differential when approaching a LV cavitary mass, including thrombus, tumors, both benign and malignant, or rarely a vegetation.

We describe a case of a 43-year-old female post AMI diagnosed with a mid-cavitary mass within the LV. Despite the use of multimodality cardiovascular imaging, she required surgical excision for the rare diagnosis of a papillary fibroelastoma.

## Case report

**Fig. 1:**
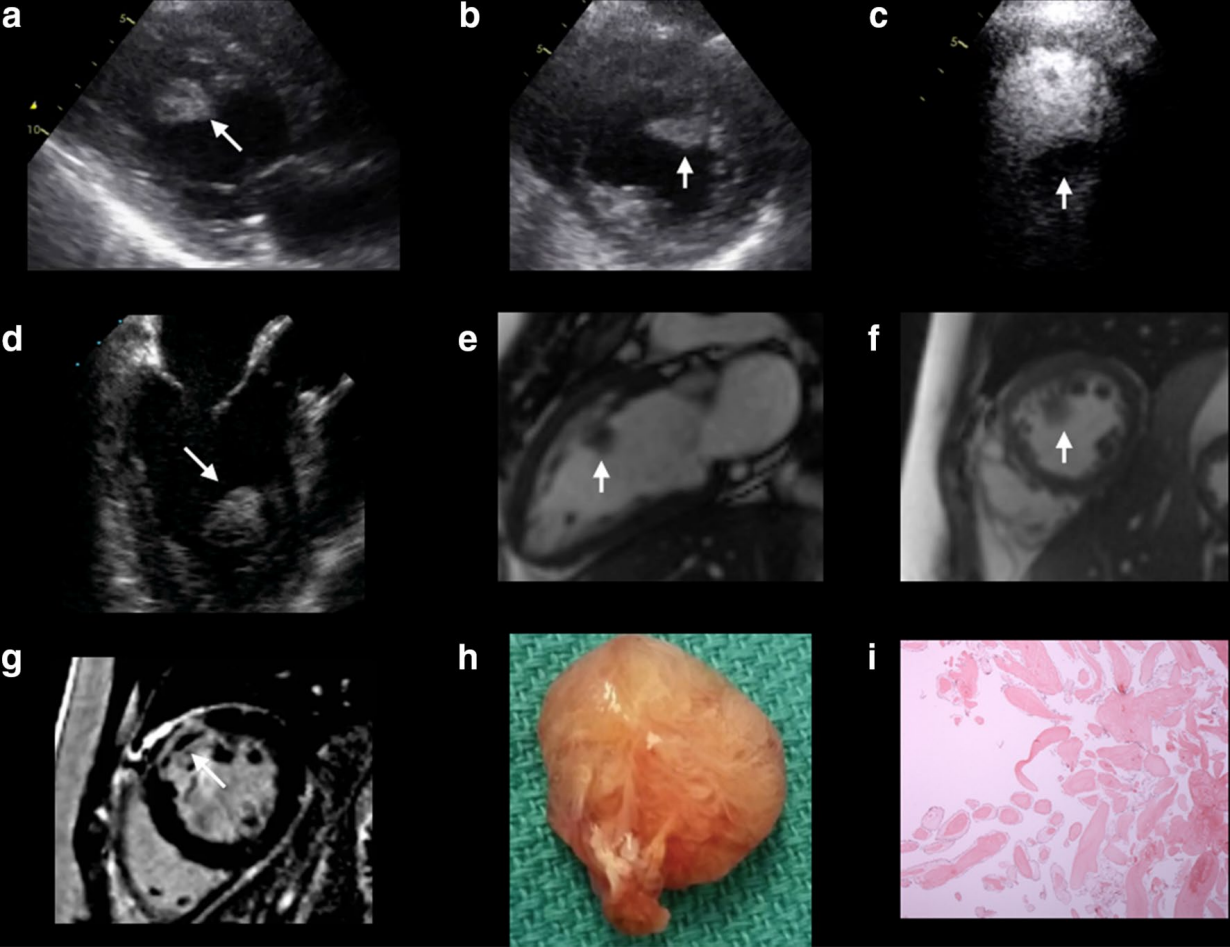
**a**, **b** A parasternal long axis and short axis view on TTE demonstrating an echodense mass (23 × 18 mm) attached to the mid anteroseptal wall of the LV; **c** An apical 4 chamber view on TTE following the administration of Definity demonstrating the avascular nature of the mass within the LV cavity; **d** A midesophageal long axis view on TEE confirming the echodense mass attached to the mid anteroseptal wall; **e**, **f** A vertical long axis and short axis view on B-SSFP imaging using CMR demonstrating the location of the intracardiac mass within the LV cavity; **g** A short axis view on DE-CMR confirming infarction of the mid to basal anterior wall; **h** Gross specimen of the LV mass (22 × 16 × 10 mm); **i** Histopathological confirmation of an avascular connective tissue with fronds covered by endothelium consistent with a papillary fibroelastoma

A 43-year-old Caucasian woman with type II diabetes, hypertension, dyslipidemia, and pre-existing ischemic heart disease (IHD), presented with a non ST-segment elevation myocardial infarction (NSTEMI). Coronary angiography demonstrated multi-vessel disease with severe in-stent restenosis of her proximal left anterior descending (LAD) and significant obstructive lesions in the proximal right coronary artery (RCA) and proximal obtuse marginal (OM) artery. Given the multi-vessel disease with two layers of stent and aggressive nature of her coronary artery disease, the patient was accepted for in-hospital coronary artery bypass grafting (CABG). A preoperative transthoracic echocardiogram (TTE) confirmed a mass (23 × 18 mm) within the LV cavity (Fig. 1a, b). Following the administration of Definity for LV opacification, the mass appeared avascular, mobile, with a broad based attachment to the mid anterior wall (Fig. 1c). Transesophageal echocardiogram confirmed the size and attachment of the LV mass to the anterior wall (Fig. 1d). Cardiac magnetic resonance imaging confirmed a LV mass measuring 1.3 cm adjacent to the akinetic mid-anterior wall (Fig. 1e, f). Following the administration of gadolinium, delayed enhancement imaging confirmed infarction of the mid to basal anterior wall without enhancement of the LV mass (Fig. 1g). As there was no evidence of late gadolinium enhancement of the LV mass on CMR, this would favor a presumed diagnosis of a LV thrombus. The patient underwent surgical resection of the mass via the aortic valve during a four vessel CABG procedure, including left internal mammary artery to LAD, left radial artery to posterior descending artery, saphenous venous graft to first obtuse marginal artery, and a second saphenous venous graft to the second obtuse marginal artery. The gross specimen of the LV mass demonstrated a 22 × 16 × 10 mm mass with a characteristic frond-like appearance, suggestive of papillary fibroelastoma (Fig. 1h). Histopathological examination confirmed an avascular connective tissue core with adjacent fronds covered by endothelium consistent with a papillary fibroelastoma (Fig. 1i).

## Discussion

Cardiac papillary fibroelastomas (PFE’s) are uncommon, benign, avascular cardiac tumors. With an estimated frequency as high as 0.33% on autopsy series, PFEs constitute up to 7% of all primary cardiac tumors [4]. PFEs are typically found on the valvular surface of the heart, most commonly involving the aortic and mitral valves [5]. Non-valvular PFEs are exceedingly rare, with only 55 patients identified in a systematic search of the literature using the MEDLINE database [6]. Although the pathophysiology of PFE is not entirely understood, there is a strong association with previous open-heart surgeries and thoracic radiation [7]. Damage to the endothelium, trauma, and organizing emboli have been suggested mechanisms [8]. Although it is a benign tumor, PFE’s can present with ischemia or severe embolic sequelae, especially when the mass arises in the left side of the heart [9]. Surgical removal of PFE can reduce the risk of embolization.

Echocardiography is the most readily and cost effective imaging modality available to assess a LV mass. It provides basic information on mass morphology, mobility, position, and attachment. Contrast echocardiography using Definity allows additional classification based on vascularity [7]. Despite its superior temporal resolution, ease of portability and real-time imaging, complete definition of cardiac masses may not be possible with echocardiography alone. Both CT and CMR allow high contrast resolution and evaluation of myocardial infiltration. CT has the benefit of delineating calcification of the mass, while CMR has the added benefits of the contrast agent gadolinium. Late gadolinium enhancement can be used to distinguish thrombi from tumor based on its lack of enhancement. It is also used to identify the presence, location and extent of an AMI [10]. Appropriate use of these various non-invasive cardiac imaging modalities often allows differentiation of the mass and a pre-operative diagnosis.

Our case presents a unique challenge to pre-operative diagnosis. Although echocardiography and CMR suggested that the mass was a thrombus, given the context of the recent anterior MI, exploration and surgical excision was completed by the surgeon due to the potential for the mass to embolize. The final diagnosis of a PFE was unique due to its unusual location and large size within the LV cavity. This unique case demonstrates shortcomings of multimodality cardiac imaging in the diagnosis of an atypical mass and the importance of obtaining tissue when clinically safe and feasible.

## Abbreviations

AMI: acute myocardial infarction
CCT: cardiac computed tomography
CMR: cardiovascular magnetic resonance
CABG: coronary artery bypass grafting
IHD: Ischemic heart disease
LV: left ventricular
NSTEMI: non ST-segment elevation myocardial infarction
OM: obtuse marginal
PFE: papillary fibroelastomas
RCA: right coronary artery
TTE: transthoracic echocardiogram
